# Reply to ‘Pitfalls in the quantitative imaging of glutathione in living cells’

**DOI:** 10.1038/s41467-018-04037-7

**Published:** 2018-04-23

**Authors:** Xiqian Jiang, Jianwei Chen, Jin Wang

**Affiliations:** 1 Department of Pharmacology and Chemical Biology, Baylor College of Medicine, Houston, TX 77030 USA; 2 Department of Molecular and Cellular Biology, Baylor College of Medicine, Houston, TX 77030 USA; 3 Center for Drug Discovery, Baylor College of Medicine, Houston, TX 77030 USA

## Introduction

Our group reported the first reversible reaction-based ratiometric probe—ThiolQuant Green—that can quantify glutathione (GSH) levels in living cells in 2015^1^. Building on this work, we developed the second generation probe, RealThiol (RT), which has a much improved reaction kinetics, quantum yield, and solubility, enabling quantitative real-time monitoring of GSH level changes in living cells^2^. Recently, we developed a mitochondria-specific GSH probe, MitoRT, which can monitor mitochondrial GSH (mGSH) dynamics^3^. The RT and MitoRT probes have generated tremendous interest in the redox biology community and been shared with more than 40 laboratories around the globe through either collaboration or kerafast.com. As one of the early adopters, the Schmidt group applied RT to monitor GSH level changes in sulfur mustard resistant keratinocytes^4^. Despite of the success of our GSH probes, each method has its own pros and cons. We appreciate Cossetti et al.’s effort to validate the RT probe and agree with their main conclusion that RT can be used as a GSH probe only when the amount of GSH is at least an order of magnitude greater than that of cysteine (Cys). Taking advantage of this opportunity, we will emphasize the limitations of the RT probe and clarify the seemingly discrepancies in Cossetti et al.’s correspondence.

The sensing mechanism of RT is based on the reversible reaction between Michael acceptors and thiols. Technically, all the thiols in cells, including GSH, Cys, and protein thiols, would react with RT. Unlike the numerous GSH probes reported^5^, our key contribution to this field is to fine tune the dissociation equilibrium constant (*K*_d_) between Michael acceptors and thiols to the mM range^6^. The benefits of the intentional design of a sensing reaction with mM *K*_d_ are two folds: i) to allow quantitation of thiols in the 1–10 mM range; and ii) to be insensitive to thiols in the µM range based on the ratiometric readouts.

**Fig. 1:**
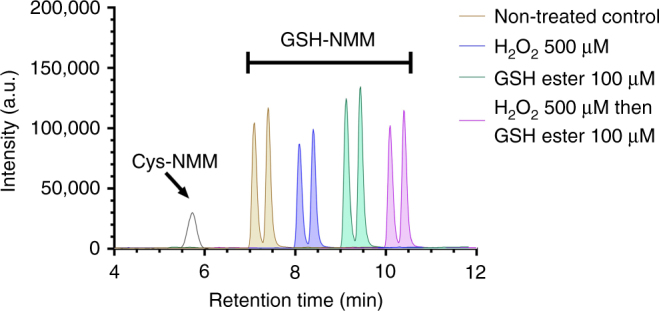
LC-MS-based quantification of GSH and cysteine in HeLa cells under different treatment conditions. After different treatments that were used in Figure 4d of ref. ^2^, cells (1 × 10^6^) were treated with 1 mM of *N*-methylmaleimide (NMM) to derivatize all the thiols and lysed in 0.5 mL of lysis buffer. Assuming the cell volume is 4000 µm^3^, the dilution factor is 125 during the lysis process. On our mass spectrometer, the lower limit of quantitation (LLOQ) for GS-NMM and Cys-NMM is 1 µM, which corresponds to an LLOQ of 125 µM for intracellular thiols. The LC-MS measured GSH levels in HeLa cells treated with PBS (shaded yellow), 500 µM of H_2_O_2_ (shaded blue), 100 µM of GSH-ethyl ester (shaded dark green), and 500 µM of H_2_O_2_ followed by 100 µM of GSH-ethyl ester (shaded purple) for 10 min are 5.0, 2.8, 5.7, and 4.4 mM, respectively. Unshaded black trace shows the chromatographic peak of Cys-NMM standard compound. However, Cys-NMM was not detected in any of these samples, indicating that the Cys levels were below 125 µM in cells and the GSH levels are at least one order of magnitude higher than the Cys levels under our experimental conditions. It should be noted that the retention times for GSH-NMM peaks under different treatment conditions (blue, green, and purple traces) were shifted by 1, 2, and 3 min, respectively, for clarity

Cossetti et al. found that RT is able to react with both GSH and Cys at 200 µM concentration. This is consistent with our data reported in Figure 2c in ref. ^2^. In Figure 2c, we demonstrated that GSH and Cys have similar reactivities toward RT. The cyan traces in Figure 2c showed that sub-mM concentration of GSH or Cys does react with a small percentage of RT, which was detected in Cossetti et al.’s HPLC experiment. However, due to the mM *K*_d_ between RT and thiols, sub-mM concentrations of thiols cause minimal changes of the ratiometric values (the blue traces in Figure 2c). Unfortunately, Cossetti et al.’s HPLC experiments (Fig. 1a, b in their correspondence) only detected the RT-thiol adducts in a non-quantitative manner and do not report on the vast majority of unreacted RT.

We agree that HPLC methods are the gold standard to quantify the concentrations of different thiol species in lysates. Cossetti et al. mentioned that “we did not show whether Cys is present or not in our HeLa cells before and after treatments with H_2_O_2_”. Indeed, we applied liquid chromatography mass spectrometry (LCMS) to check the GSH and Cys levels and found GSH is the dominant thiol species under our experimental conditions (Fig. 1). In Cossetti et al.’s Fig. 1d, it appeared that the levels of GSH and Cys are comparable (in the µM range) in HeLa cells treated with 200 µM of H_2_O_2_. However, the treatment time in Cossetti et al.’s experiment was 24 h, which is much longer than our treatment time of 10 min. In our own hands, most of the HeLa cells become dead with 200 µM of H_2_O_2_ treatment for 24 h.

In Cossetti et al.’s Fig. 1c, the levels of Cys and GSH in mouse hippocampus are almost identical, 110 and 125 µM, respectively. We do not have data to compare with this measurement. Hippocampus is a highly complexed organ with at least 47 subclasses of cells^7^. HPLC based bulk measurements cannot reflect the GSH and Cys levels in single cells and may be complicated by the interstitial fluid, which contains high levels of Cys^8^. In contrast to Cossetti et al.’s result, Wang and Cynader reported the GSH level is at least 10 times of the Cys level in astrocytes and neurons^9^. Nonetheless, under conditions where the Cys and GSH levels are ~100 µM in cells, RT should not be used since the concentrations are out of its dynamic range.

Cossetti et al. also point out that 1 mM of NMM we used was “insufficient to be sure that all thiols present are blocked, especially in cells where GSH levels are higher than 1 mM”. It should be noted that 100 µM of NMM in the experimental section of ref. ^2^ is a typo, which should be corrected to 1 mM. We did not choose 10 mM of NMM for thiol quenching because cells die almost immediately if 10 mM of NMM is added to the medium, which makes it difficult to perform subsequent assays. NMM has limited aqueous solubility. Excessive NMM precipitates out from the solution, resulting in low apparent concentration. Considering that the volume of culture medium is orders of magnitude larger than that of all the cells in each dish, 1 mM of NMM is sufficient to quench all the intracellular thiols. Even if 1 mM of NMM did not completely react with all the thiols, the ratio for GSH-NMM and Cys-NMM adducts would remain the same because the reactivities for GSH and Cys toward Michael acceptors are almost identical. In our LC-MS experiments, we only detected GSH-NMM but not Cys-NMM, indicating that the GSH level is at least an order of magnitude higher than that of Cys under our experimental conditions (Fig. 1).

Cossetti et al. mentioned the reversibility of the reaction between thiols and maleimides to justify the use of high concentration of maleimides. Based on Baldwin and Kiick’s study, the half-life of retro-Michael addition for an alkyl thiol and a maleimide is over 300 h^10^, suggesting that the retro-Michael addition reaction is not a serious concern if the samples are measured shortly after cell lysis.

Despite the advantages of RT, it is important to understand its limitations to obtain meaningful measurements. The sensing moiety of RT essentially reacts with all the small molecule thiols, including GSH, Cys, and even reduced trypanothione, but to a lesser extent toward protein thiols, probably due to steric hindrance or charge interactions. RT can be claimed as a GSH probe only when GSH is the dominant form of small molecule thiols and in the mM range. In the case that the total concentration of small molecule thiols is in the µM range, RT cannot perform accurate measurements since it is outside the dynamic range. If Cys were in the mM range in cells, RT would respond to Cys as well, although we are unaware of a situation like this in mammalian cells. Therefore, we advise users to either measure relative concentrations of Cys and GSH in the system they use by HPLC methods or search for available literature data before using the RT probe. We hope this fruitful discussion would clarify confusions and guide future applications of RT.

## Data availability

All relevant data are available from the authors upon request.

## References

[1] Jiang X (2015). Quantitative imaging of glutathione in live cells using a reversible reaction-based ratiometric fluorescent probe. ACS Chem. Biol..

[2] Jiang X (2017). Quantitative real-time imaging of glutathione. Nat. Commun..

[3] Chen J (2017). Reversible reaction-based fluorescent probe for real-time imaging of glutathione dynamics in mitochondria. ACS Sens..

[4] Rothmiller, S. et al. Sulfur mustard resistant keratinocytes obtained elevated glutathione levels and other changes in the antioxidative defense mechanism. *Toxicol. Lett*. 10.1016/j.toxlet.2017.11.024. (2017). 10.1016/j.toxlet.2017.11.024 PMC6235149 29183814

[5] Yin CX, Xiong KM, Huo FJ, Salamanca JC, Strongin RM (2017). Fluorescent probes with multiple binding sites for the discrimination of Cys, Hcy, and GSH. Angew. Chem..

[6] Jiang, X. et al. Challenges and opportunities for small molecule fluorescent probes in redox biology applications. *Antioxid. Redox Signal*. 10.1089/ars.2017.7491 (2018). 10.1089/ars.2017.7491 PMC6056262 29320869

[7] Zeisel A (2015). Brain structure. Cell types in the mouse cortex and hippocampus revealed by single-cell RNA-seq. Science.

[8] Bito L, Davson H, Levin E, Murray M, Snider N (1966). The concentrations of free amino acids and other electrolytes in cerebrospinal fluid, in vivo dialysate of brain, and blood plasma of the dog. J. Neurochem..

[9] Wang XF, Cynader MS (2000). Astrocytes provide cysteine to neurons by releasing glutathione. J. Neurochem..

[10] Baldwin AD, Kiick KL (2011). Tunable degradation of maleimide-thiol adducts in reducing environments. Bioconjug. Chem..

